# Importance of Internet Surveillance in Public Health Emergency Control and Prevention: Evidence From a Digital Epidemiologic Study During Avian Influenza A H7N9 Outbreaks

**DOI:** 10.2196/jmir.2911

**Published:** 2014-01-17

**Authors:** Hua Gu, Bin Chen, Honghong Zhu, Tao Jiang, Xinyi Wang, Lei Chen, Zhenggang Jiang, Dawei Zheng, Jianmin Jiang

**Affiliations:** ^1^Zhejiang Provincial Center for Disease Control and PreventionHangzhouChina; ^2^Department of Public HealthCollege of Health and Human ServicesWestern Kentucky UniversityBowling Green, KYUnited States

**Keywords:** influenza A virus, H7N9 subtype, Internet, surveillance, disease outbreak

## Abstract

**Background:**

Outbreaks of human infection with a new avian influenza A H7N9 virus occurred in China in the spring of 2013. Control and prevention of a new human infectious disease outbreak can be strongly affected by public reaction and social impact through the Internet and social media.

**Objective:**

This study aimed to investigate the potential roles of Internet surveillance in control and prevention of the human H7N9 outbreaks.

**Methods:**

Official data for the human H7N9 outbreaks were collected via the China National Health and Family Planning Committee website from March 31 to April 24, 2013. We obtained daily posted and forwarded number of blogs for the keyword “H7N9” from Sina microblog website and a daily Baidu Attention Index (BAI) from Baidu website, which reflected public attention to the outbreak. Rumors identified and confirmed by the authorities were collected from Baidu search engine.

**Results:**

Both daily posted and forwarded number and BAI for keyword H7N9 increased quickly during the first 3 days of the outbreaks and remained at a high level for 5 days. The total daily posted and forwarded number for H7N9 on Sina microblog peaked at 850,000 on April 3, from zero blogs before March 31, increasing to 97,726 on April 1 and to 370,607 on April 2, and remaining above 500,000 from April 5-8 before declining to 208,524 on April 12. The total daily BAI showed a similar pattern of change to the total daily posted and forwarded number over time from March 31 to April 12. When the outbreak locations spread, especially into other areas of the same province/city and the capital, Beijing, daily posted and forwarded number and BAI increased again to a peak at 368,500 and 116,911, respectively. The median daily BAI during the studied 25 days was significantly higher among the 7 provinces/cities with reported human H7N9 cases than the 2 provinces without any cases (*P*<.001). So were the median daily posted and forwarded number and daily BAI in each province/city except Anhui province. We retrieved a total of 32 confirmed rumors spread across 19 provinces/cities in China. In all, 84% (27/32) of rumors were disseminated and transmitted by social media.

**Conclusions:**

The first 3 days of an epidemic is a critical period for the authorities to take appropriate action through Internet surveillance to prevent and control the epidemic, including preparation of personnel, technology, and other resources; information release; collection of public opinion and reaction; and clarification, prevention, and control of rumors. Internet surveillance can be used as an efficient and economical tool to prevent and control public health emergencies, such as H7N9 outbreaks.

##  Introduction

Human infections with new avian influenza A (H7N9) virus have been reported in Shanghai, Anhui, Zhejiang, and Jiangsu provinces in China beginning March 31, 2013 [1]. A total of 108 cases confirmed with DNA sequencing and 23 deaths of H7N9 infections have been reported until April 24, 2013. This is the first time that human infections with H7N9 virus have been found and reported in the world [1]. Cases were mainly distributed in provinces in the southeast of China. Almost 64% cases had exposure to animals or visited a live poultry market [2]. Clinical symptoms and signs were of sudden onset, including respiratory symptoms in the early stage, high fever (≥ 38°C) and cough, dyspnea after 5 to 7 days onset, and then severe progressive pneumonia. Some cases could rapidly develop acute respiratory distress syndrome and die [2,3]. This outbreak has received great public attention from all over the world. Its progress has been continuously reported by various media in China.

The Internet has been developed and popularized quickly in China in the past decade. According to the China Internet Development Statistical Report, there were 538 million Internet users (39.9% of the whole population) and 388 million cell phone Internet users by June 30, 2012 [4]. Internet users in China spend, on average, nearly 20 hours per week online [4]. They now have access to news and information through more channels including microblogs and social networking websites. In addition, they can expand news coverage by sharing and forwarding information online. Now there are millions of social media users and the number is increasing [4]. Sina microblog has the largest number of users in China, approximately 368 million users at the end of 2012. Web search engines are an important tool for Internet users to acquire information. In China, there are approximately 429 million users using search engine websites, among which Baidu website has the largest group of users [4]. In 2011, 77.2% Internet users preferred Baidu website as their first search engine [5]. The power of Internet users in China is becoming stronger, especially in public opinion leadership with increases in participation for big events. Reactions from Internet users can roughly represent public reactions to an event. Developed Internet and social media provide an excellent chance to understand public reactions to and clearly communicate about public health emergencies, including preparation, prevention, and control of emergent infectious disease outbreaks [6-8].

In recent years, China has experienced increasing outbreaks of emerging infectious diseases. The severe acute respiratory syndrome (SARS) outbreaks, epidemics, and pandemics in 2003 and influenza A H1N1 outbreaks in 2009 have led to strong repercussions among the public and media. The public and media reactions during an outbreak period are a double-edged sword. Moderate reaction may arouse individuals’ awareness of disease control and prevention, whereas overreaction could play a negative role in the population. During the period of SARS pandemics, there were network media information chaos, rumors, and public panic, including overbuying of medical-related products and the soaring prices of these products, which destabilized the society and caused many problems against disease control and prevention [9]. Control and prevention of emerging infectious disease outbreaks require public participation. How Internet users react to an emergent disease outbreak and how we can lead them to a proper response are extremely important for control and prevention of the disease in the population. During the H7N9 outbreak, we designed a study to investigate the potential roles of Internet surveillance on reactions of Internet users to the H7N9 outbreaks and provide evidence for government, health authorities, and the public to efficiently control and prevent public health emergency problems, such as H7N9, in the future.

## Methods

### Study Design and Population

#### Overview

We designed and conducted an exploratory digital epidemiologic study of public reaction to the outbreaks of human infection with H7N9 virus from March 31 to April 24, 2013. Our study population was Internet users in China during the study period of Avian Influenza A H7N9 outbreaks.

#### Case Sources

The first human case with H7N9 virus infection was reported by the China National Health and Family Planning Committee (CNHFPC) on March 31, 2013. We collected and analyzed data from the first 25 days of the outbreak (March 31 to April 24, 2013) as our study period because the government stopped daily case reports after April 24, 2013. All the reported cases and deaths were obtained from the website of the CNHFPC [10]. All cases were confirmed by DNA sequencing.

#### Internet Surveillance of Public Reactions

Since the H7N9 outbreak, we initiated Internet surveillance of Internet users’ reactions to it. Crowdsourcing [11] was used as an approach to collect data from millions of Internet users in Sina microblog and the Baidu website. Using the keyword “H7N9,” we collected the daily tally of relevant blogs posted and forwarded (daily posted and forwarded number) in Sina microblog from March 31 to April 24, 2013. We also obtained the daily posted and forwarded number for H7N9 in 9 provinces/cities, of which 7 provinces/cities (Shanghai, Anhui, Jiangsu, Zhejiang, Beijing, Henan, and Shandong) had reported H7N9 cases and 2 provinces (Hubei and Shaanxi) had no cases. The daily posted and forwarded number can be used as an estimate for the degree of attention to an event from the public. The daily posted and forwarded numbers in the 2 provinces without cases were used as a base/reference. These numbers were officially released by Sina microblog website [12].

The daily Baidu Attention Index (BAI) was used as another data source. The daily BAI computes the weighted sum of searching frequency for a keyword based on its daily search volume on the Baidu website [13]. The daily BAI for the keyword H7N9 was collected from the BAI webpage from March 31 to April 24, 2013 [13]. Meanwhile, the daily BAI for the keyword H7N9 from the 7 provinces that reported human H7N9 cases were also collected during the study period. The daily BAI data from Hubei and Shaanxi provinces in the Midwest of China, which had no human H7N9 cases reported, were collected as a base/reference to compare the differences of public reactions with the provinces with H7N9 cases.

In addition, we used the keywords “H7N9” and “rumor” to search for rumors of H7N9 that were officially verified and confirmed by the local authorities on the Baidu website. A total of 32 rumors were collected during the study period. We then categorized the rumors based on their characteristics. All the information obtained online was in simplified Chinese language and released publicly by the websites, but no personal identification information, such as name or email address, was collected. This study was approved by the Institutional Review Board in the Zhejiang Provincial Centers for Disease Control and Prevention.

### Statistical Analysis

We graphed the curves of H7N9 epidemic and severity by number of daily cases, first case(s) reported in each province/city, and cumulative case fatality rate over time. The daily posted and forwarded number and BAI were graphed by date, number of daily cases, and first case(s) in each province/city, respectively, to explore public reaction changing to the H7N9 epidemic situation over time. Median (P_50_) and range were used to describe the distributions of daily posted and forwarded number and BAI indexes by province/city. Spearman’s rank correlation coefficient was used to check the relationship of epidemic trends with the daily posted and forwarded number and BAI, respectively, if the distribution of the variables is monotonic. Kruskal-Wallis test was used to test the differences in daily posted and forwarded number and BAI between provinces/cities with cases and without cases. Rumors of H7N9 outbreaks were qualitatively classified by date, province, rumor maker, media, and main content.

## Results

### H7N9 Epidemic Trend

**Figure 1:**
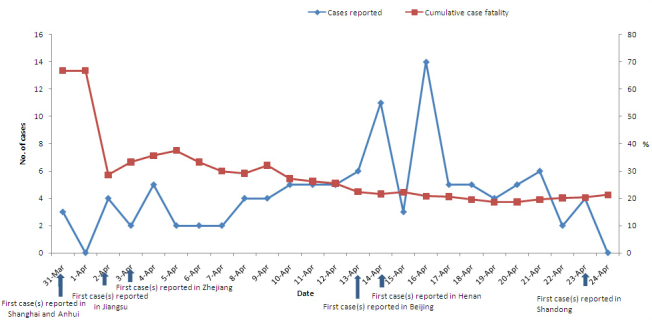
The human cases of avian influenza A H7N9 virus infection reported with the first case(s) by province/city and the cumulative fatality rate in China from March 31 to April 24, 2013.

On March 31, 2013, the CNHFPC reported the first human infections with the H7N9 virus, including 2 cases in Shanghai and 1 in Anhui province [10]. Subsequently, cases were successively reported in Jiangsu, Zhejiang, Beijing, Henan, and other provinces. Until April 24, 2013, a total of 108 cases and 23 deaths (cumulative case fatality rate = 21.3%) of H7N9 infections were reported in 7 provinces/cities in China [10]. The cumulative case fatality rate was 33% (6/18) during the first week and then gradually declined to 21.3% (23/108) because of timely prevention and treatment. By April 12, 43 cases had been reported in 4 provinces in Eastern China and then the epidemic expanded to Beijing on April 13 and Henan province on April 14. The last case during the study period was reported in Shandong province on April 23, 2013 (Figure 1).

### Daily Posted and Forwarded Number for H7N9 on Sina Microblog

**Figure 2:**
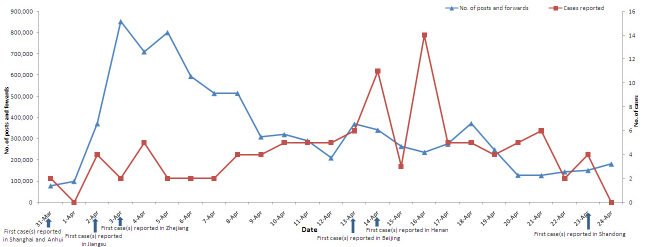
The reported human H7N9 cases and the daily posted and forwarded number of H7N9 discussion trends on Sina microblog in China from March 31 to April 24, 2013.

**Figure 3:**
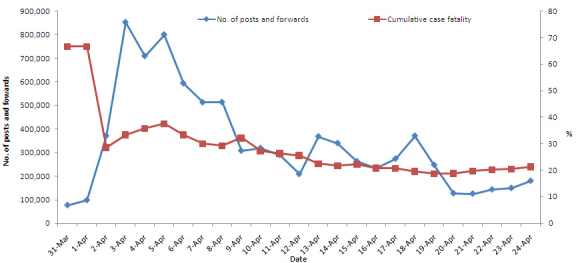
The daily posted and forwarded number of H7N9 discussion trends on Sina microblog positively associated with cumulative case fatality rate of human H7N9 infection in China from March 31 to April 24, 2013.

The total daily posted and forwarded number for the keyword H7N9 on Sina microblog peaked at 850,000 on April 3, 2013, from zero before March 31, increasing to 97,726 on April 1, and to 370,607 on April 2, and remaining above 500,000 from April 5 to 8 before declining to 208,524 on April 12. When the first case was reported in Capital Beijing on April 12 and the first case in Henan province on April 14, the total daily posted and forwarded number returned to a relatively high level of 368,500 on April 13 and 339,822 on April 14, declined to 262,667 on April 15 and 235,131 on April 16, and increased again to 273,752 on April 17 and 370,995 on April 18. The total daily posted and forwarded number then decreased and remained at a level of 100,000 (Figure 2). The median of the total daily posted and forwarded number was 289,325 (range 76,482-853,027). Spearman’s rank correlation coefficient analyses showed that the daily posted and forwarded number was positively associated with cumulative case fatality rate (Spearman’s rank correlation coefficient = 0.60, *P*=.002), that is to say, higher cumulative case fatality rate higher daily posted and forwarded number at the early stage of epidemic and lower cumulative case fatality rate lower daily posted and forwarded number later (Figure 3).

**Figure 4:**
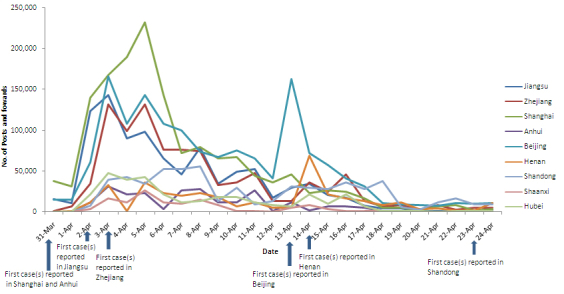
The daily posted and forwarded number for H7N9 on Sina microblog by province/city from March 31 to April 24, 2013.

Since the first human H7N9 case reported on March 31, the observed daily posted and forwarded number in each province/city showed a similar trend to the total daily posted and forwarded number. However, the daily posted and forwarded number in Beijing on April 14 and Henan on April 15 rose to another peak when the local area first reported cases (the daily posted and forwarded number peaked at 163,512 in Beijing on April 13 and peaked at 72,512 in Henan province on April 14 as shown in Figure 4).

The observed median daily posted and forwarded number during the 25 days studied was 36,256 (range 1830-232,368) in Shanghai, 4944 (217-31,312) in Anhui, 21,424 (1521-143,376) in Jiangsu, 24,720 (897-131,840) in Zhejiang, 57,680 (7867-166,448) in Beijing, 11,536 (316-56,032) in Henan, 28,016 (513-56,032) in Shandong, 9888 (327-47,792) in Hubei, and 924 (229-26,368) in Shaanxi. The mean rank value of the daily posted and forwarded number in each province/city with human H7N9 cases reported except Anhui was significantly higher (*P*<.001) than that in the 2 provinces without cases (Table 1).

**Table 1:** The observed median daily posted and forwarded number and Baidu Attention Index (BAI) for H7N9 by province/city on Sina microblog and Baidu website from March 31 to April 24, 2013.

Province/city	Daily posted and forwarded number^a^		Daily BAI^b^	
	Median	Mean rank value	Median	Mean rank value
Shanghai	36,256	150.26	4227	144.16
Anhui	4944	70.68	1723	62.16
Jiangsu	21,424	133.96	6592	175.68
Zhejiang	24,720	134.04	6432	174.66
Beijing	57,680	163.18	4790	148.60
Henan	11,536	97.08	2731	108.82
Shandong	28,016	127.54	3065	112.96
Hubei^c^	9888	88.92	1420	51.06
Shaanxi^c^	924	51.34	1301	38.90

^a^Kruskal-Wallis test of daily posted and forwarded number: χ^2^_8_=67.5 (*P*<.001).

^b^Kruskal-Wallis test of daily BAI: χ^2^_8_=129.2 (*P*<.001).

^c^Provinces without cases reported as base/reference.

### Daily Baidu Attention Index for H7N9

**Figure 5:**
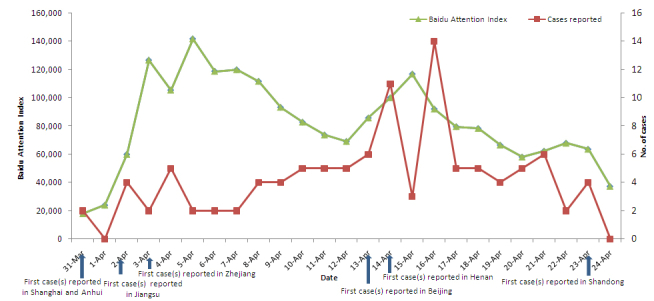
The reported human H7N9 cases and the daily Baidu Attention Index (BAI) of H7N9 discussion trends on Baidu website in China from March 31 to April 24, 2013.

**Figure 6:**
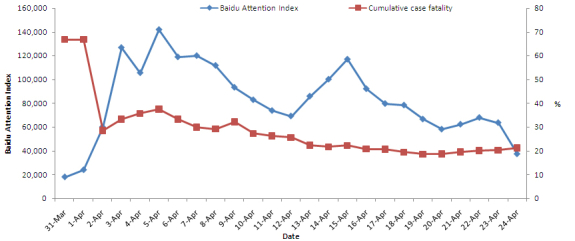
The daily Baidu Attention Index (BAI) of H7N9 discussion trends on Baidu website positively associated with cumulative case fatality rate of human H7N9 infection in China from March 31 to April 24, 2013.

The daily BAI for keyword H7N9 increased sharply in the first 3 days of the H7N9 outbreak, with a peak at 126,825 on April 3. The daily BAI then declined with fluctuations, remaining at a high level above 100,000 from April 4 - 8. Following the cases reported in Beijing and Henan, the daily BAI increased again and peaked at 116,991 on April 15. Afterwards, it steadily decreased to a lower level of approximately 40,000. The median daily BAI was 79,687 (range 18,007-79,687) (Figure 5). Spearman’s rank correlation coefficient analyses showed that the daily BAI was positively associated with cumulative case fatality rate (Spearman’s rank correlation coefficient = 0.43, *P*=.04), that is to say, higher cumulative case fatality rate higher daily BAI at the early stage of epidemic and lower cumulative case fatality rate lower daily BAI later (Figure 6).

The daily BAI for H7N9 in each province/city increased substantially after the first human cases were reported regardless of where human cases were reported. The trend of daily BAI change in each province/city was similar to the total daily BAI. Moreover, there was also an obvious increase of the daily BAI in Beijing and Henan from April 14-15 because of the first case report in Beijing on April 13 and in Henan on April 14 and in Shandong province when the government first announced a case on April 23.

Overall, the median daily BAI was 4227 (range 0-9014) in Shanghai, 1723 (0-2765) in Anhui, 6592 ( 0-11,914) in Jiangsu, 6432 (0-15,011) in Zhejiang, 4790 (0-9488) in Beijing, 2731 (0-7592) in Henan, 3065 (0-6763) in Shandong, 1420 (0-2707) in Hubei, and 1301 (0-2198) in Shaanxi. The mean rank values of daily BAI in the provinces with reported cases were significantly higher than that in the provinces without cases (*P*<.001; Table 1).

**Figure 7:**
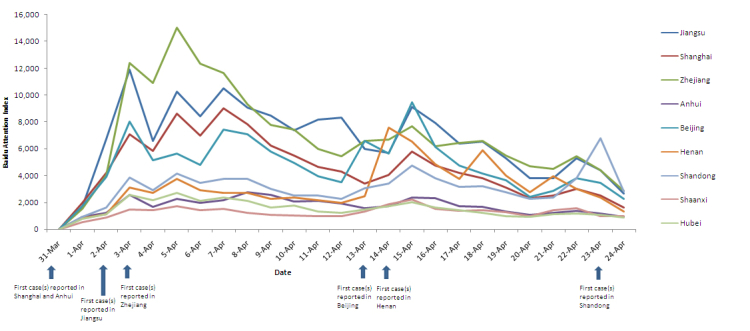
Daily Baidu Attention Index (BAI) by province/city in China from March 31 to April 24, 2013.

### Rumors of H7N9 Outbreaks

**Figure 8:**
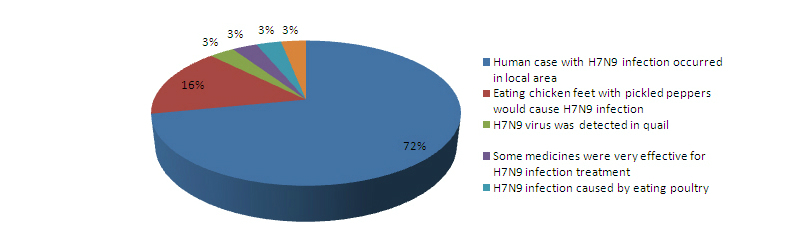
A total of 32 confirmed rumors for H7N9 outbreaks classified by main content during the H7N9 outbreaks in China from March 31 to April 24, 2013.

All 32 rumors were disseminated by 58 people and distributed in 19 provinces in China. In all, 84% (27/32) rumors were disseminated through Sina microblog, QQ (the most popular Chinese communication software), and Wechat (an instant mobile communication software), and 16% (5/32) were spread via bulletin board system (BBS), cell phone message, and telephone calls. Of these 27 rumors, 72% (23/32) disseminated information that human infections with H7N9 virus occurred in the local areas, and 16% declared that eating chicken feet with pickled peppers (a popular Chinese food) would cause H7N9 infection (Figure 8). Among 15 rumor investigations that released the characteristics of the rumor makers, 5 were students; 5 were migrant workers; and 5 were farmers and company employees. Four rumor investigations announced the aims for making and disseminating rumors were to obtain more attention or get more visits to their individual forum or microblog. Some rumors had very strong transmissibility. The rumor that “there was a suspected human case with H7N9 virus infection in a local school” disseminated by a student from Gansu province, Western China, was forwarded by 34,000 people. The rumor that “eating chicken feet with pickled peppers would cause H7N9 infection” was spread across 5 provinces in China.

## Discussion

### Principal Findings

Our study used Internet surveillance to investigate the public reaction to the H7N9 outbreaks over time during the first 25 days of the H7N9 epidemic in China in the spring of 2013. The indexes of public reaction, daily posted and forwarded number and BAI, to H7N9 outbreaks were significantly higher in 7 provinces/cities with human H7N9 cases than those in 2 provinces without cases. Both daily posted and forwarded number and BAI were positively associated with the cumulative case fatality rate of human H7N9 infection. Our findings indicate that the first 3 days of an epidemic is a critical period for the authorities to take appropriate responsible action through Internet surveillance to control and prevent the epidemic, including preparation of personnel, technology, and other resources; information release; collection of public opinion and reaction; and clarification, prevention, and control of rumors. Internet surveillance can be used as an efficient and economical tool to control and prevent public health emergencies, such as H7N9 outbreaks.

This is the first study that investigated the importance of infoveillance [7,14-17] methods in monitoring public reaction to the H7N9 avian influenza outbreak on Sina microblog and Baidu search engines and to have collected rumors through the Internet in China. When an emerging infectious disease outbreak occurs, it is important for risk communicators in the government to clearly know the public concern [18]. In the past, to obtain information on the public, community, and media reactions or concern, most researchers used questionnaires and telephone interviews after the outbreak [19-23]. In contrast, network monitoring can be timelier to know public reactions to the outbreak because it is a real-time surveillance during the emergencies and also it is very economical without consuming too many resources [6,7,13,16,24,25]. During this human H7N9 avian influenza outbreak, we obtained the information by Internet monitoring and provided timely policy suggestions to the governments and related shareholders who play a role in disease outbreak control and prevention.

Emerging infectious diseases usually have the features of unknown pathogens and high mortality, no vaccine or effective treatment available, and quick and wide dissemination that trigger high public and media attention [18,26]. As soon as the government announced the outbreak, the public would have concern about the event and be eager to find relevant information to obtain a certain sense of safety [22,25]. Our study suggested that the first 3 days of the outbreak was the period when Internet attentions rose rapidly. At this point, the government needs to timely and transparently release the information, including personal protective methods of the disease, which can ease public tension quickly [18]. During this human H7N9 outbreak, when the first human case was announced, the authorities timely released case numbers and disease prevention knowledge online, which may be one of the key reasons for the early decline of public attention. However, the high attention to this outbreak lasted 3 - 5 days, which is a good time for the Department of Emergency Control and Prevention to disseminate health education knowledge and skills through network and social media.

Some studies have shown that the public concern with the H1N1 influenza infection was associated with the number of reported cases and hospitalizations by the governments [22,23]. However, other studies reported that although the number of flu cases rose, the tweets about the H1N1 influenza showed a downward trend [16]. This fact reflected that public concern or interests for the outbreak might be weakened along with the progress of the epidemic as information saturation sets in [16,27]. Our study showed public reaction was positively associated with the cumulative case fatality rate but not daily number of cases. When case fatality rate was high, the public attention was high. The public attention decreased with a decrease in case fatality rate. This indicates that the public attention decreases when the threat from the epidemic disease decreases. Our study showed a decreasing trend of Internet user’s attention during the middle and later period. However, when there were new cases reported in a new province/city, the daily posted and forwarded number and BAI increased significantly, especially in the local province/city. This phenomenon indicates that the extension of epidemic area triggered more public attention than the increase of reported cases in the original outbreak areas. It is necessary to monitor the public reaction and take specific responses at that moment.

Rumors generate and occur early during the period of public health emergency [28-30]. The rumors of human H7N9 outbreaks collected in this study covered most provinces in China, and also spread quickly. Most of the rumors claimed that H7N9 avian influenza cases occurred in the local areas. The uncertainty of emerging infectious diseases often generates the rumors, which also suggests the importance of early information release [19,25,31-32]. In this new age of social media, microblogs are easy to generate and spread rumors, yet they could also be used to clarify wrong information and stop the dissemination.

###  Limitations

There were some limitations in this study. The daily posted and forwarded number and BAI were collected from the Internet and could not be standardized because of lack of exact numbers of daily Internet users. Thus, the mean rank of daily posted and forwarded number and BAI only provide relative comparisons between provinces/cities with and without H7N9 cases, but there is not much meaning to comparisons among individual provinces/cities. Our study was limited to the first 25 days of the outbreak because the government stopped daily case reports, instead using weekly case reports; therefore, this might not thoroughly reflect public reaction after the first 25 days. Our study primarily focused on Chinese websites, which limits the representativeness of the public reaction from Internet users on English and other language websites. Our study is an exploratory study that was designed to use a quick way to obtain public reaction during the study period of a public health emergency. The qualitative research on rumors was simple and did not use frameworks to analyze because of the diversity of the information released on the Internet. The results of our study could be applicable to public health emergency response, but may not be directly applied to public health practice yet. Further studies with advanced methods or study designs are needed.

### Conclusions

This exploratory digital epidemiologic study provides evidence that Internet surveillance is a rapid and efficient way to evaluate public reactions, which will help prepare the government, health authorities, and the public to respond to public health emergencies. Our findings showed the public reaction increased quickly during the early stage of the outbreak. The first 3 days could be a “golden 3 days” for the government and public health authorities to release information in time and make the information transparent and open. The high public attention would last for a week; thus, this week could be a critical period for health education. Expansion of the disease into other areas has more impact on public attention than the number of cases reported in the same outbreak area. This implies that extra methods should be taken when the epidemic area is expanded. Rumors are always hard to avoid, but using media, such as releasing information via an official microblog, could be effective to quickly clarify rumors and prevent their further spread.

## Abbreviations

BAI: Baidu Attention Index
SARS: severe acute respiratory syndrome
CNHFPC: China National Health and Family Planning Committee
